# Role of Sox2 in Learning, Memory, and Postoperative Cognitive Dysfunction in Mice

**DOI:** 10.3390/cells10040727

**Published:** 2021-03-24

**Authors:** Lingli Gui, Zhen Luo, Weiran Shan, Zhiyi Zuo

**Affiliations:** 1 Department of Anesthesiology, University of Virginia, Charlottesville, VA 22901, USA; gui_lingli@hotmail.com (L.G.); pippa922@163.com (Z.L.); sw8gm@virginia.edu (W.S.); 2 Department of Anesthesiology, Tongji Hospital, Tongji Medical College, Huazhong University of Science and Technology, Wuhan 430030, China; 3 Department of Anesthesiology, West China Hospital, Sichuan University, No. 37 Guo Xue Alley, Chengdu 610041, China

**Keywords:** cognitive enrichment, environmental enrichment, mice, postoperative cognitive dysfunction, sex-determining region Y-box-2

## Abstract

Postoperative cognitive dysfunction (POCD) is a significant clinical issue. Its neuropathogenesis has not been clearly identified and effective interventions for clinical use to reduce POCD have not been established. This study was designed to determine whether environmental enrichment (EE) or cognitive enrichment (CE) reduces POCD and whether sex-determining region Y-box-2 regulated by sirtuin 1, plays a role in the effect. Eighteen-month-old male mice were subjected to right-common-carotid-artery exposure under sevoflurane anesthesia. Some of them stayed in cages with EE or CE after the surgery. Learning and memory of mice were tested by a Barnes maze and fear conditioning, starting 2 weeks after the surgery. Sex-determining region Y-box-2 (Sox2) in the brain was silenced by small hairpin RNA (shRNA). Immunofluorescent staining was used to quantify Sox2-positive cells. Surgery reduced Sox2-positive cells in the hippocampus (64 ± 9 cells vs. 91 ± 9 cells in control group, *n* = 6, *p* < 0.001) and impaired learning and memory (time to identify target box one day after training sessions in the Barnes maze test: 132 ± 53 s vs. 79 ± 53 s in control group, *n* = 10, *p* = 0.040). EE or CE applied after surgery attenuated this reduction of Sox2 cells and POCD. Surgery reduced sirtuin 1 activity and CE attenuated this reduction. Resveratrol, a sirtuin 1 activator, attenuated POCD and surgery-induced decrease of Sox2-positive cells. Silencing shRNA reduced the Sox2-positive cells in the hippocampus and impaired learning and memory in mice without surgery. These results suggest a role of Sox2 in learning, memory, and POCD. EE and CE attenuated POCD via maintaining Sox2-positive cells in the hippocampus.

## 1. Introduction

More than 50 million Americans have surgery each year. Post-operative cognitive dysfunction (POCD) is a documented clinical phenomenon that is attracting significant attention from the public and professionals in recent years [1,2,3,4]. POCD is characterized by decline of learning and memory after surgery. POCD can be associated with an increased mortality [2,5]. Advanced age has been consistently identified as a risk factor for the delayed phase of POCD [1,2]. About 10% of the elderly (60 years or older) patients have POCD 3 months after non-cardiac surgery [2]. This issue is very significant because elderly patients are more likely to have surgery and it will become even more significant in the future because of an increase in the elderly population [6,7]. However, suitable interventions for clinical use to reduce POCD have not been established.

Environmental enrichment (EE) has been shown to improve cognitive functions [8,9,10]. We and others have shown that EE reduced post-surgery learning and memory impairment in mice [11,12], suggesting that EE is a potential non-pharmacological method to reduce POCD. However, the EE condition used in the previous studies may significantly enhance physical activity [11,12]. It may be difficult for elderly patients to have significant physical activity immediately after surgery. It is not known yet whether facilitating cognitive activity after surgery will have any effect on POCD. In addition, the mechanism for the protection of EE against POCD is not fully determined.

Neuroplasticity is the underlying structural process for learning and memory [13]. The plasticity includes cellular changes and brain cell genesis. Generation of brain cells is from local stem cells and progenitor cells that are maintained in certain areas of the adult brain, one of which is the dentate gyrus of hippocampus [14]. Sex-determining region Y-box-2 (Sox2) is a transcription factor that is critical for maintaining pluripotency of stem cells [15]. Sox2 also regulates the expression of multiple genes that are critical for neuronal differentiation and maturation [16]. Although studies have shown that various insults impair learning and memory and reduce Sox2 expression [17,18,19], it is not known whether there is a causal relationship between the impaired learning and memory and decreased Sox2 expression.

We hypothesize that surgery reduces Sox2 expression, which contributes to POCD. To address this hypothesis, Sox2 was silenced in wild-type mice or maintained after surgery by activating sirtuin 1 (Sirt1), also known as NAD-dependent deacetylase sirtuin-1, which can regulate Sox2 expression [20].

## 2. Materials and Methods

The animal protocol was approved by the institutional Animal Care and Use Committee of the University of Virginia (Charlottesville, VA, USA). All animal experiments were carried out in accordance with the National Institutes of Health’s Guide for the Care and Use of Laboratory Animals (NIH publications number 80–23) revised in 2011.

### 2.1. Animal Groups

**Figure 1:**
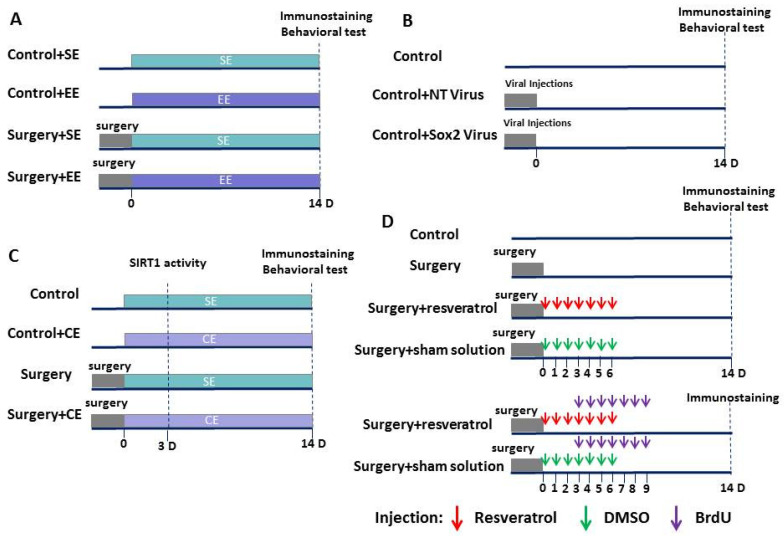
Schematic presentations of experiments. (**A**) Four groups of mice were studied—control plus standard environment (SE), control plus environmental enrichment (EE), surgery plus SE, and surgery plus EE. Brain tissues were collected at 14 d after surgery for immunostaining. Barnes maze and fear-conditioning tests were performed at 14 d after surgery. (**B**) Three groups of mice were studied—control, non-targeting small hairpin RNA (shRNA) lentivirus (control + non-targeting (NT) virus) and sex-determining region Y-box-2 (Sox2) shRNA lentivirus (control + Sox2 virus). Brain tissues were collected at 14 d after surgery for immunostaining. Fourteen days after surgery, Barnes maze and fear-conditioning tests were performed. (**C**) Four groups of mice were studied—control, cognitive enrichment (CE), surgery plus SE, and surgery plus CE. Brain tissues were collected at 3 d after surgery for activating sirtuin 1 (Sirt1) activity measurement, and were harvested 14 d after surgery for immunostaining. Fourteen days after surgery, Barnes maze and fear-conditioning tests were performed. (**D**) Four groups of mice were studied—control, surgery, surgery plus resveratrol, and surgery plus sham solution. Mice in the surgery plus resveratrol or surgery plus sham solution groups received daily intraperitoneal injection of resveratrol or sham solution, respectively. This injection was started 2 h after the surgery and was for 7 days. Brain tissues were collected at 14 d after surgery for immunostaining. Barnes maze and fear-conditioning tests were performed 14 d after surgery. Another two groups of mice, surgery plus resveratrol or surgery plus sham solution, received seven consecutive intraperitoneal injections of 5′-bromo-2′-deoxyuridine (BrdU) once daily. These mice were sacrificed 5 days later for immunostaining.

In the first experiment, eighteen-month-old male C57BL/6 mice weighing 26–38 g from National Institute on Aging (Bethesda, MD, USA) were randomly assigned to four groups—control (mice were not exposed to anesthesia and surgery), EE (mice were exposed to EE but were not exposed to anesthesia and surgery), surgery plus standard environment (SE), and surgery plus EE. In the second experiment, mice were assigned to three groups—control, non-targeting (NT) shRNA lentivirus, and Sox2 shRNA lentivirus, (Figure 1A,B). All mice before surgery or EE were housed under SE with three to four mice per cage in a 27.94 × 15.24 × 11.43 cm cage on a 12-h light/dark cycle with ad libitum access to food and water. Mice were then kept under this SE or an EE starting from day 0 after the surgery. For those mice that were assigned to surgery plus EE, surgery was finished before 12:00 on that day so that they could be placed in EE starting at 18:00. EE was performed as previously described [11]—three to four mice were placed in a large cage (cage size 43.18 × 22.86 × 19.05 cm) for 15 h (18:00 to 9:00 the next day) each day. The cage contained a running wheel, tunnels, shed, and various toys. These settings were changed twice per week. The animals were housed under SE during the rest of the time each day. These housing environments were maintained until the animals had completed the behavioral tests (*n* = 10 for each group) or were sacrificed for brain tissue harvest (*n* = 6 for each group).

In the third experiment, 18-month-old male C57BL/6 mice weighing 32–38 g were randomly assigned to four groups—control, cognitive enrichment (CE), surgery plus SE, and surgery plus CE (Figure 1C). In the fourth experiment, mice were randomly assigned to control, surgery, surgery plus resveratrol, and surgery plus sham solution (Figure 1D). CE was performed as previously described [11,21]—mice were placed in a large cage (cage size: 43.18 × 22.86 × 19.05 cm) for 6 h from 12:00 to 18:00 each day. The cage contained many toys of different shapes, sizes, colors, and textures, but did not contain specific facilitators like tunnels, ladders, ropes, or running wheels that could increase physical activity. These settings were changed twice a week. Mice were housed under SE during the rest of time each day. These housing environments were maintained until the animals had completed the behavioral tests (*n* = 8–11 for each group) or were sacrificed for brain tissue harvest (*n* = 6–8 for each group).

We did not establish exclusion criteria. Data from all animals that completed the assigned study were included in the analysis.

### 2.2. Anesthesia and Surgery

The surgery was a right carotid artery exposure [22]. Briefly, mice were anesthetized by 2.5% sevoflurane (the first and second experiments) or 1.5% isoflurane (the third and fourth experiments) in oxygen. During the procedure, spontaneous respiration was maintained and rectal temperature was monitored and maintained at 37 °C with the aid of a heating blanket (TCAT-2LV, Physitemp Instruments Inc., Clifton, NJ, USA). A 1.5-cm midline neck incision was made after the mouse was exposed to sevoflurane or isoflurane for at least 30 min. Soft tissues over the trachea were retracted gently. One-centimeter-long right common carotid artery was carefully dissected free from adjacent tissues without any damage on the vagus nerve. The wound was then irrigated and closed by using surgical suture. The surgical procedure was performed under sterile conditions and lasted approximately 10 min. After the surgery, all animals received a subcutaneous injection of 3 mg/kg bupivacaine. The total duration of anesthesia was 2 h. No response to toe pinching was observed during the anesthesia. Our previous studies had shown that animals maintained good arterial blood oxygen saturation and heart rates under these surgery and anesthesia conditions [23,24].

### 2.3. Learning and Memory Testing

Learning and memory were evaluated by the Barnes maze and fear-conditioning tests. These tests were conducted from 14:00 to 16:00 in a sound-isolated room. After these tests the mice were not used for any biochemistry studies and were sacrificed with deep anesthesia.

### 2.4. Barnes Maze

Fourteen days after being exposed to various experimental conditions, animals were subjected to the Barnes maze to test their spatial learning and memory as previously described [22]. The Barnes maze is a circular platform with 20 equally spaced holes (SD Instruments, San Diego, CA, USA). One of the holes was connected to a dark chamber that was called the target box. The test was started by placing animals in the middle of the Barnes maze. Aversive noise (85 db) and bright light from a 200-W bulb shed on platform were used to provoke mice to find and enter the target box. The animal would be kept in the target box for 1 min after entering the box. If the animal was unable to find the target box, it was removed from the platform and placed in the target box for 1 min. Each animal was observed for up to 3 min at a time. Animals were trained in a spatial acquisition phase that took 4 days with 3 min per trial, four trials per day, with a 15-min interval between each trial. The memory test was carried out on day 5 (short-term retention) and day 12 (long-term retention). No test was performed during the period from day 5 to day 12. The latency to enter the target box during each trial was recorded and analyzed by an ANY-Maze video tracking system (SD Instruments, San Diego, CA, USA).

### 2.5. Fear Conditioning

A fear-conditioning test was performed 24 h after the Barnes maze test as previously described [22]. Briefly, it consisted of three phases—the training (acquisition), the context-related test, and the tone-related test. In the training phase, mice were placed in a test chamber and received three tone–foot shock pairings (tone: 2000 Hz, 85 db, 30 s; shock: 0.7 mA, 2 s) with a 1-min interval in a relatively dark room. The chamber was then cleaned with 70% ethanol. In the context-related test, mice were placed in the same chamber without any stimulation for 8 min. The duration of freezing behavior was recorded. In the tone-related test conducted two hours later, mice were placed in a new test chamber for 7.5 min in a relatively light room. This chamber had a different context and smell from the first test chamber and was wiped with 1% acetic acid. No auditory stimulus was provided in the first 3 min of this session. Three 30-s tone stimulus cycles with 1 min interval were carried out in the last 4.5 min. The freezing behavior in this 4.5 min period was recorded. Both context- and tone-related tests were recorded by a video recording system. Freezing behavior recorded in the video was scored by an observer blinded to group assignment.

### 2.6. Sox2 shRNA and Lentiviral Vector Construction

Ten different *Sox2* target sequences were predicted according to the RNAi designer (https://rnaidesigner.invitrogen.com/rnaiexpress/, accessed on 20 March 2021). After comparing and analyzing with previous studies [25,26], codes for shRNA targeting at the sequences 778–798 were chosen to be engineered to the pcDNA^TM^6.2-GW/miR shRNA expression vector (BLOCK-iT Pol II miR RNAi Expression Vector Kit, Invitrogen, Carlsbad, CA, USA). Sox2 shRNA forward sequence: 5′-TGCTGAAGAAGGATAAGTACACGCTTGTTTTGGCCACTGACTGACAAGCGTGTTTATCCTTCTT-3′, Sox2 shRNA reverse sequence: 5′-CCTGAAGAAGGATAAACACGCTTGTCAGTCAGTGGCCAAAACAAGCGTGTACTTATCCTTCTTC-3′. The non-targeting shRNA sequence was as described before [27], and did not target any known eukaryotic gene (5′-AAATGTACTGCGCGTGGAGACGTTTTGGCCACTGACTGACGTCTCCACGCAGTACATTT-3′). The sequences were verified by sequencing.

shRNAs in the pcDNA^TM^ 6.2-GW/miR expression vector were then subcloned into pLenti6/V5-DEST Gateway Vector (Invitrogen) to generate Sox2 shRNA and non-targeting shRNA constructs. 293FT cells were transfected with pLenti6/V5-DEST by lipofectamine 2000 (catalogue number: 11668019, Invitrogen) to produce lentivirus as instructed by the protocol from Invitrogen. High-titer pseudo-typed lentiviral particles were harvested by PEG-it™ Virus Precipitation Solution (catalogue number: LV810A-1, System Biosciences, Palo Alto, CA, USA). Titers were 1.34 × 10^10^ (Sox2 shRNA) and 2 × 10^9^ (non-targeting shRNA) transducing units/mL measured by qPCR lentivirus titration kit (Applied Biological Materials Inc., Richmond, BC, Canada). The viruses were then aliquoted and kept at −80 °C until use.

### 2.7. Viral Injections

In the second experiment, some mice received bilateral intracerebroventricular injection of 3 μL NT shRNA lentivirus or Sox2 shRNA lentivirus. Each mouse received only one injection to each ventricle under sevoflurane anesthesia. The intracerebroventricular injection was performed as described before [28,29] with the aid of a stereotactic apparatus (SAS-5100, ASI Instruments, Warren, MI, USA) using the following coordinates: 1 mm posterior to bregma, 1.3 mm lateral from midline and 3 mm ventral from the surface of the skull. Lentivirus was injected at the rate of 1 μL/min. After the injection, the needle was kept in place for 1 min to prevent backflow of the injected solution.

### 2.8. Drug Administration

In the fourth experiment, some mice received daily intraperitoneal injection of 50 mg/kg resveratrol (Abcam, Cambridge, UK; dissolved in 25% DMSO to a final concentration of 10 mg/mL) based on a previous study [30] and some mice received intraperitoneal injection of the DMSO solvent (sham solution). This injection was started 2 h after the surgery and was for 7 days.

### 2.9. 5′-bromo-2′-deoxyuridine (BrdU) Administration

Two days after surgery, mice were given seven consecutive intraperitoneal injections of 100 mg/kg 5′-bromo-2′-deoxyuridine (Sigma-Aldrich, St Louis, MO, USA) at 17:00 once daily as previously described [11,31]. Mice were sacrificed 5 days later for immunostaining.

### 2.10. Brain Tissue Harvesting

Mice were deeply anesthetized with isoflurane at 3 days (for measuring Sirt1 activity), 14, or 28 days (for immunostaining) after the surgery. They were perfused with normal saline. A coronal brain slice between Bregma -1 mm to -3 mm was harvested for immunofluorescent staining. These slices containing hippocampus were fixed with 4% paraformaldehyde. For Sirt1 activity measurement, the hippocampus and cerebral cortex were dissected. All dissection procedures were performed on ice.

### 2.11. Immunofluorescent Staining

Antigen retrieval of 5 µm thick coronal sections was performed by incubating the sections with sodium citrate buffer containing 10 mM sodium citrate and 0.05% Tween 20 (pH 6.0) at 95 to 100 °C for 20 min. DNA denaturation was performed by incubating with 1 N HCL at 4 °C for 3 min, 2 N HCL at room temperature for 3 min, and then at 37 °C for 6 min. Sections were blocked with 5% normal donkey serum and 1% bovine serum albumin in Tris-buffered saline containing 0.5% triton-X 100 for 2 h at room temperature. The sections were then incubated overnight at 4 °C with the following primary antibodies: goat polyclonal anti-Sox-2 antibody (1:100 dilution, catalogue number: sc-17320; Santa Cruz Biotechnology, Santa Cruz, CA, USA), rabbit polyclonal anti-glial fibrillary acidic protein (GFAP) antibody (1:1000 dilution, catalogue number: ab7260; Abcam), rabbit polyclonal anti-doublecortin antibody (1:100 dilution, catalogue number: ab18723; Abcam), rabbit monoclonal anti-NeuN antibody (1:100 dilution, catalogue number: ab177487; Abcam), or rat monoclonal anti-BrdU antibody (1:100 dilution, catalogue number: ab6326; Abcam). The sections were incubated with donkey anti-goat IgG antibody conjugated with Alexa Fluor 594 (1:200 dilution, catalogue number: A-11058; Invitrogen), donkey anti-rabbit IgG antibody conjugated with Alexa Fluor 488 (1:200 dilution, catalogue number: A-21206; Invitrogen), or donkey anti-rat IgG antibody conjugated with Alexa Fluor 594 (1:200 dilution, catalogue number: ab150156; Abcam) for 1 h at room temperature in a dark room. After being washed in phosphate-buffered saline, sections were counterstained with Hoechst 33,342 (Thermo Scientific, Waltham, MA), rinsed and mounted with Vectashield mounting medium (H-1000; Vector Labs, Burlingame, CA, USA). For each mouse brain, six sequential hippocampal sections were used for cell counting. The number of all cells positively stained for an interesting marker or the combination of two markers in the dentate gyrus of each section was counted to derive the average of the positively stained cell numbers (method used by Lingli Gui for experiments 1 and 2). The quantification of cell numbers for experiments 3 and 4 were done by Zhen Luo in the following way. Nine sequential hippocampal sections were used for cell counting. The number of all cells positively stained for an interesting marker or the combination of two markers in the area surrounded by the two granular layers of the dentate gyrus in each section was counted to derive the average of the positively stained cell numbers. The determination of cell numbers was done in a blind fashion.

### 2.12. Sirt1 Activity

Freshly harvested hippocampus and cerebral cortex were homogenized by 1 mL ice-cold lysis buffer (10 mM TrisHCl pH 7.5 containing 10 mM NaCl, 15 mM MgCl_2_, 250 mM sucrose, 0.5% NP-40, and 0.1 mM ethylene glycol tetraacetic acid (EGTA)). After being kept on ice for 15 min, the homogenates were spun through 4 mL of sucrose cushion containing 30% sucrose, 10 mM Tris HCl (pH 7.5), 10 mM NaCl, and 3 mM MgCl_2_ at 1300× *g* for 10 min at 4 °C. The supernatant was discarded, and the nuclear pellet was washed with cold 10 mM Tris-HCl pH 7.5 containing 10 mM NaCl. The nuclei were suspended in 100 μL of extraction buffer containing 50 mM HEPES KOH (pH 7.5), 420 mM NaCl, 0.5 mM disodium ethylenediaminetetraacetate dehydrate, 0.1 mM EGTA, and 10% glycerol, then sonicated for 30 sec and were kept on ice for 30 min. After being centrifuged at 20,000× *g* for 10 min, the supernatant (crude nuclear extract) was ready for use. Sirt1 activity was measured using a deacetylase fluorometric assay kit (SIRT1 Fluorometric Assay Kit, catalogue number: ab156065, Abcam) following the manufacturer’s protocol. After the addition of fluorosubstrate peptide, the fluorescence intensity was immediately detected using a microtiter plate fluorometer with excitation at 340 nm and emission at 460 nm at 2-min intervals for a total of 60 min. Sirt1 deacetylase activity was normalized by the protein concentrations. The results are reported as arbitrary fluorescence units (AFU).

### 2.13. Statistical Analysis

Results are presented as means ± S.E.M. (*n* ≥ 6). Data from the training sessions of the Barnes maze were analyzed by one-way (for comparisons within a group) or two-way (for comparisons between groups) repeated measures analysis of variance followed by Tukey’s test to determine the difference within the same group or between groups, respectively. The other data were analyzed by one-way analysis of variance followed by Tukey’s test with normally distributed data or by one-way analysis of variance on ranks followed by the Tukey test with non-normally distributed data. Differences were considered significant at a *p* < 0.05. All statistical analyses were performed with SigmaStat (Systat Software, Inc., Point Richmond, CA, USA).

### 2.14. Data Sharing Plan

All data and materials will be provided upon request according to established policy by the National Institute of Health, Bethesda, MD, USA.

## 3. Results

### 3.1. EE Attenuated Surgery-Induced Impairment of Learning and Memory and Reduction of Sox2-Positive Cells

**Figure 2:**
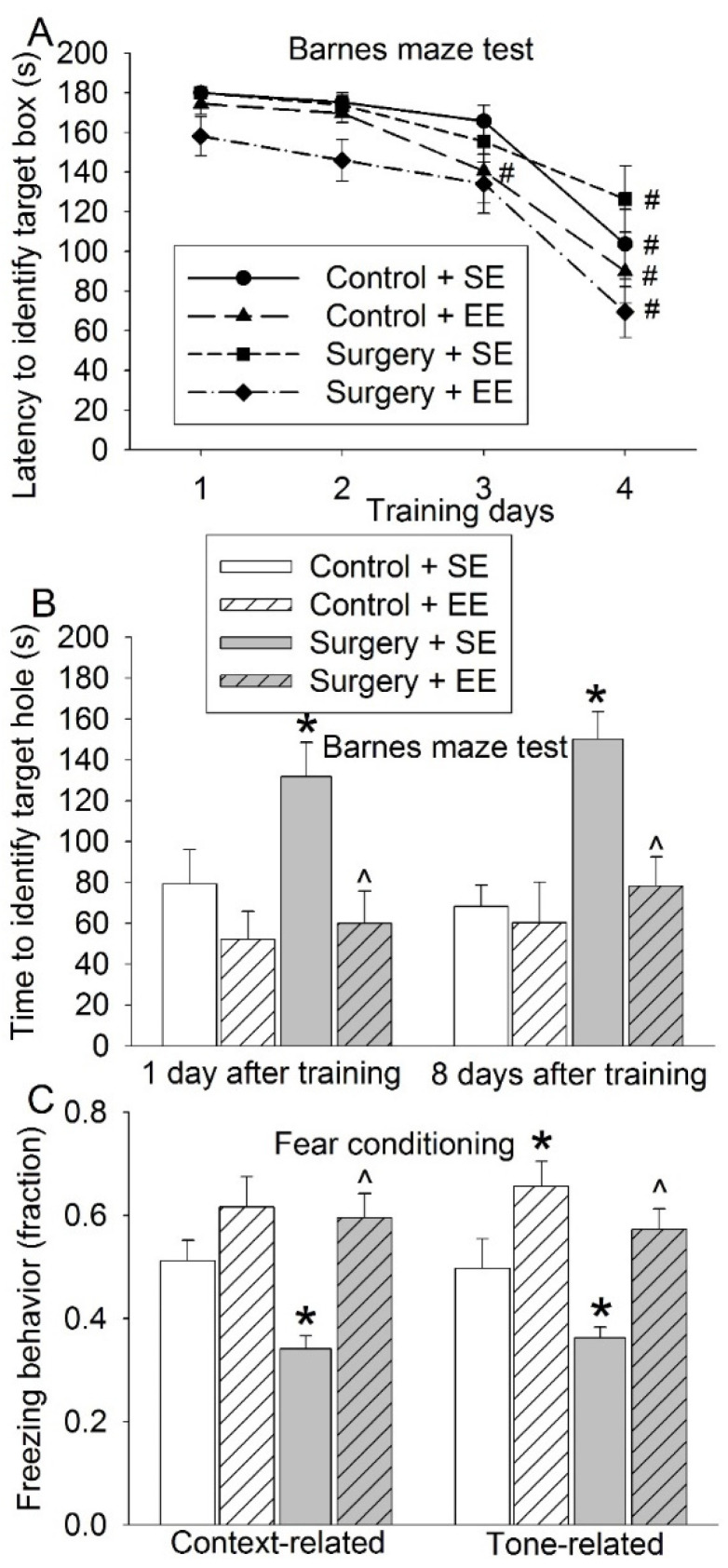
Effects of environmental enrichment and surgery on learning and memory. Eighteen-month-old mice were subjected to right carotid artery exposure and environmental enrichment. They were starting to be tested by the Barnes maze and fear conditioning 2 weeks after surgery. (**A**) Performance during the training sessions of the Barnes maze test. (**B**) Performance during the memory phase of the Barnes maze test. (**C**) Performance in the fear-conditioning test. Results are means ± S.E.M. (*n* = 10). # *p* < 0.05 compared with the corresponding data on day 1, * *p* < 0.05 compared with control, ^ *p* < 0.05 compared with surgery group.

Mice in all four groups took less time to identify the target box with increased training sessions in the Barnes maze. The time needed for the mice to identify the target box on day 4 was shorter than that on day 1 (Figure 2A). These results suggest a spatial learning process in these mice. Although EE was not a significant factor in affecting the time to identify the target box during training sessions in mice without surgery (F(1,18) = 1.298, *p* = 0.270), EE significantly affected this time in mice with surgery (F(1,18) = 8.470, *p* = 0.009). Surgery significantly increased the time for the mice to identify the target box one day and 8 days after the training sessions. EE reversed this increase (Figure 2B). Mice with surgery had reduced freezing behavior in the context- and tone-related fear conditioning. EE attenuated this decrease (Figure 2C). These results suggest that surgery induces learning and memory impairment and that EE attenuates this impairment.

**Figure 3:**
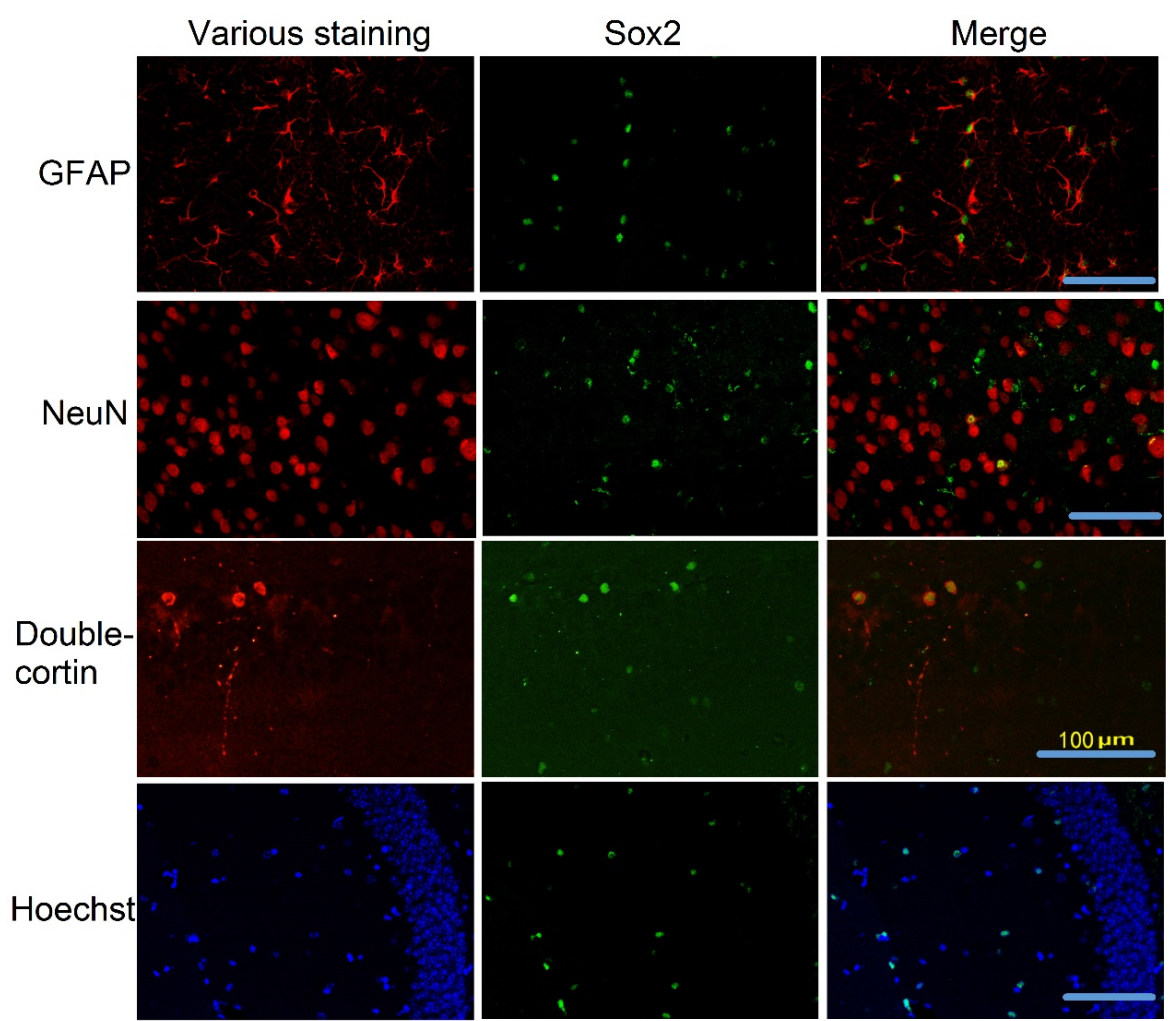
Representative images of co-staining of Sox2 with various proteins in the hippocampal dentate gyrus of control mice. Scale bar: 100 µm.

Brain cell genesis is an important structural process for learning and memory [32]. Brain cell genesis in the dentate gyrus includes neurogenesis and astrogenesis [32]. Consistent with this knowledge, many Sox2-positive cells were positively stained for GFAP, a marker for astrocytes [33]. Some Sox2-positive cells were positively stained for doublecortin, a marker for neuronal precursors and immature neurons [34], and NeuN, a marker for mature neurons [35]. Consistent with its function as a transcription factor [15], Sox2 mainly existed in the nuclei and peri-nuclear area because Sox2 staining overlapped well with the staining of NeuN and Hoechst, both of which shall stain nuclei (Figure 3)

**Figure 4:**
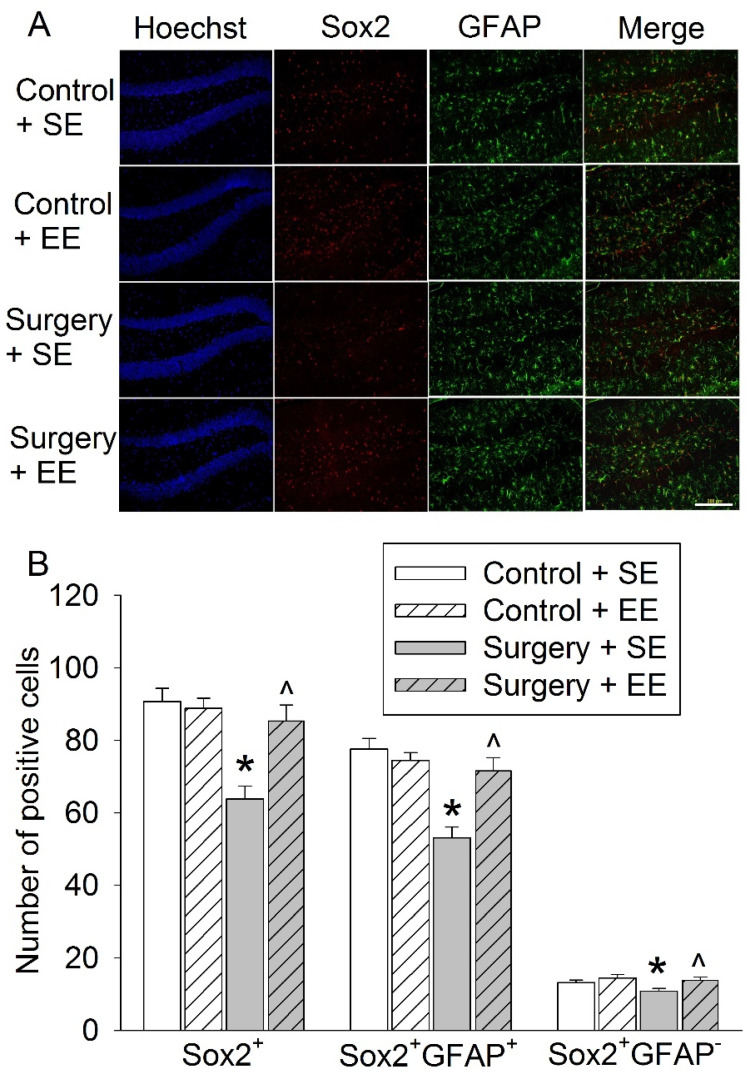
Effects of environmental enrichment and surgery on Sox2 expression in the hippocampus. Eighteen-month-old mice were subjected to right carotid artery exposure and environmental enrichment. Their brains were harvested 4 weeks after surgery. (**A**) Representative images of hippocampal dentate gyrus. Scale bar: 200 µm. (**B**) Quantitative results. Results are means ± S.E.M. (*n* = 6). * *p* < 0.05 compared with control, ^ *p* < 0.05 compared with surgery group.

Mice with surgery had decreased Sox2-positive cells in the hippocampus. The majority of the Sox2-positive cells were glial fibrillary acidic protein (GFAP) positive. Surgery also decreased Sox2-positive and GFAP-negative cells. EE blocked these surgery effects (Figure 4A,B). These results suggest that surgery reduces stem cells with pluripotency and that EE preserves these cells

### 3.2. Sox2 Silencing Impaired Learning and Memory

**Figure 5:**
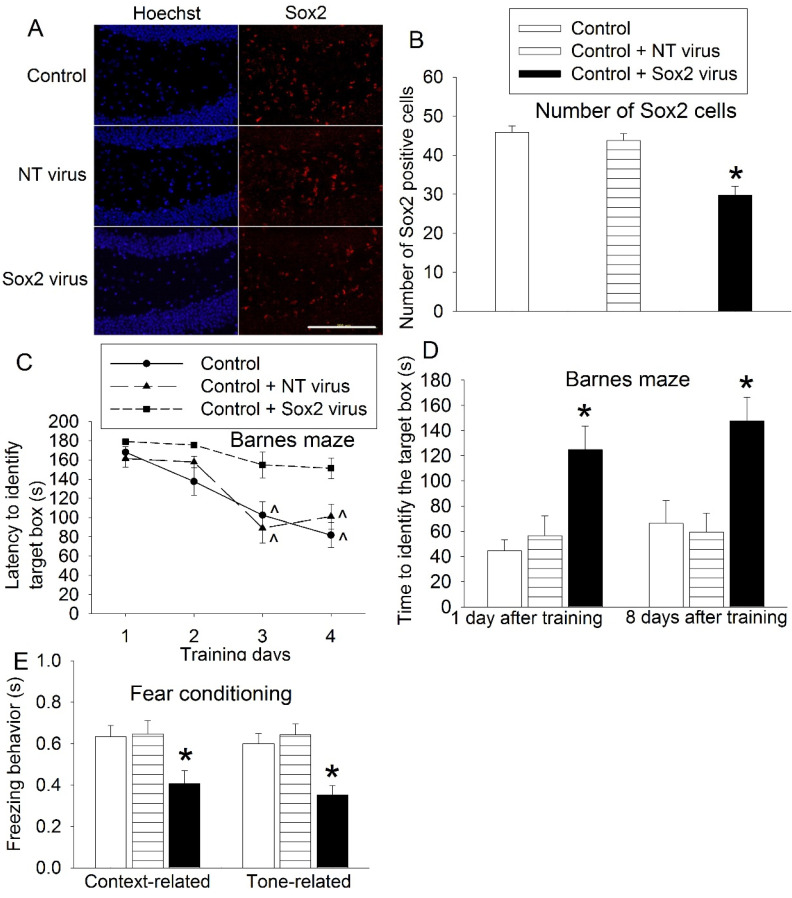
Effects of silencing Sox2 on learning and memory. Eighteen-month-old mice received shRNA to silence Sox2. Two weeks after they received shRNA, they started to be tested by the Barnes maze and fear conditioning. (**A**) Representative images of hippocampal dentate gyrus. Scale bar: 200 µm. (**B**) Quantification data of Sox2-positive cells. (**C**) Performance during the training sessions of the Barnes maze test. (**D**) Performance during the memory phase of the Barnes maze test. (**E**): Performance in the fear-conditioning test. Results are means ± S.E.M. (*n* = 6 for panel B, 10 for panels C, D and E). ^ *p* < 0.05 compared with the corresponding data on day 1, * *p* < 0.05 compared with control. NT: non-targeting.

To determine the role of Sox2 in learning and memory, Sox2 was silenced by shRNA. Virus carrying code for Sox2 shRNA was able to reduce Sox2-positive cells in the hippocampus; whereas the virus carrying code for a non-targeting shRNA fragment was not able to change the number of Sox2-positive cells (Figure 5A,B). Control mice and mice that received virus with non-targeting shRNA code had reduced time to identify the target box with increased training sessions in the Barnes maze tests but this phenomenon was not observed in the mice that received virus carrying Sox2 shRNA code. Receiving virus-carrying Sox2 shRNA code was a significant factor to affect the time to identify the target box during the training sessions of the Barnes maze (F(1,18) = 14.149, *p* = 0.001) (Figure 5C). In addition, mice receiving this virus had increased time to identify the target box one day and 8 days after the training sessions in the Barnes maze test and reduced freezing behavior in the context- and tone-related fear conditioning. Consistent with the Sox2 expression data, mice receiving virus carrying non-targeting shRNA code did not have a change in the performance of the Barnes maze and fear-conditioning tests (Figure 5D,E). These results suggest a critical role of Sox2 in learning and memory.

### 3.3. CE Attenuated Surgery-Induced Impairment of Learning and Memory and Reduction of Sox2-Positive Cells and Sirt1 Activity

**Figure 6:**
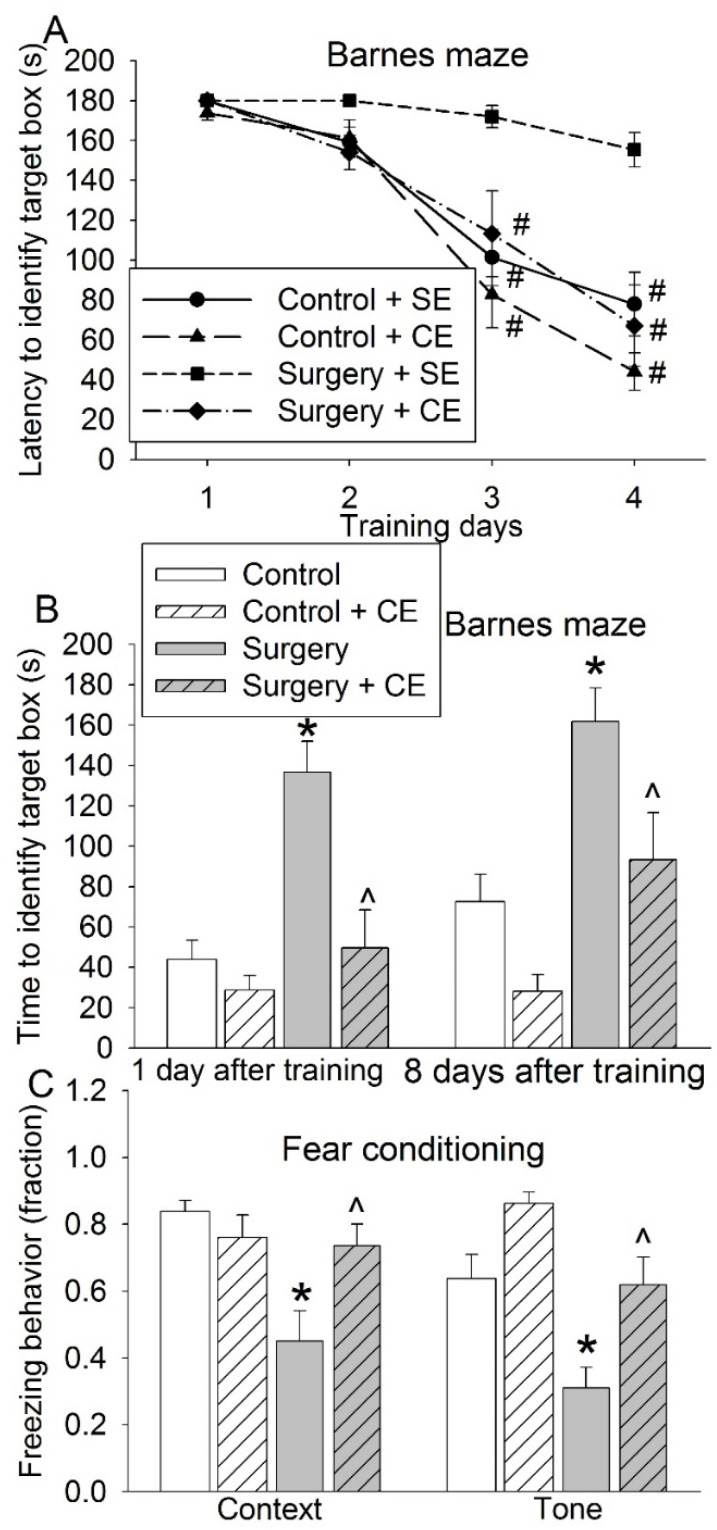
Effects of cognitive enrichment and surgery on learning and memory. Eighteen-month-old mice were subjected to right carotid artery exposure and cognitive enrichment. They were starting to be tested by the Barnes maze and fear conditioning 2 weeks after surgery. (**A**) Performance during the training sessions of the Barnes maze test. (**B**) Performance during the memory phase of Barnes maze test. (**C**) Performance in the fear-conditioning test. Results are means ± S.E.M. (*n* = 8–10). # *p* < 0.05 compared with the corresponding data on day 1, * *p* < 0.05 compared with control, ^ *p* < 0.05 compared with surgery group.

EE include physical activity that may be performed by some elderly patients, we subjected old mice to CE in which an increase in physical activity was not a major component. Surgery significantly affected the time for mice to identify the target box in the training sessions of the Barnes maze (F(1,17) = 22.389, *p* < 0.001). This surgical effect was affected by CE (F(1,16) = 16.774, *p* < 0.001) (Figure 6A). CE also attenuated the surgery-induced increase of time for mice to identify the target box one day or 8 days after the training sessions in the Barnes maze test and a decrease in the freezing behavior in the fear-conditioning test (Figure 6B,C). These results suggest that CE, similar to EE, also reverses surgery-induced impairment of learning and memory.

**Figure 7:**
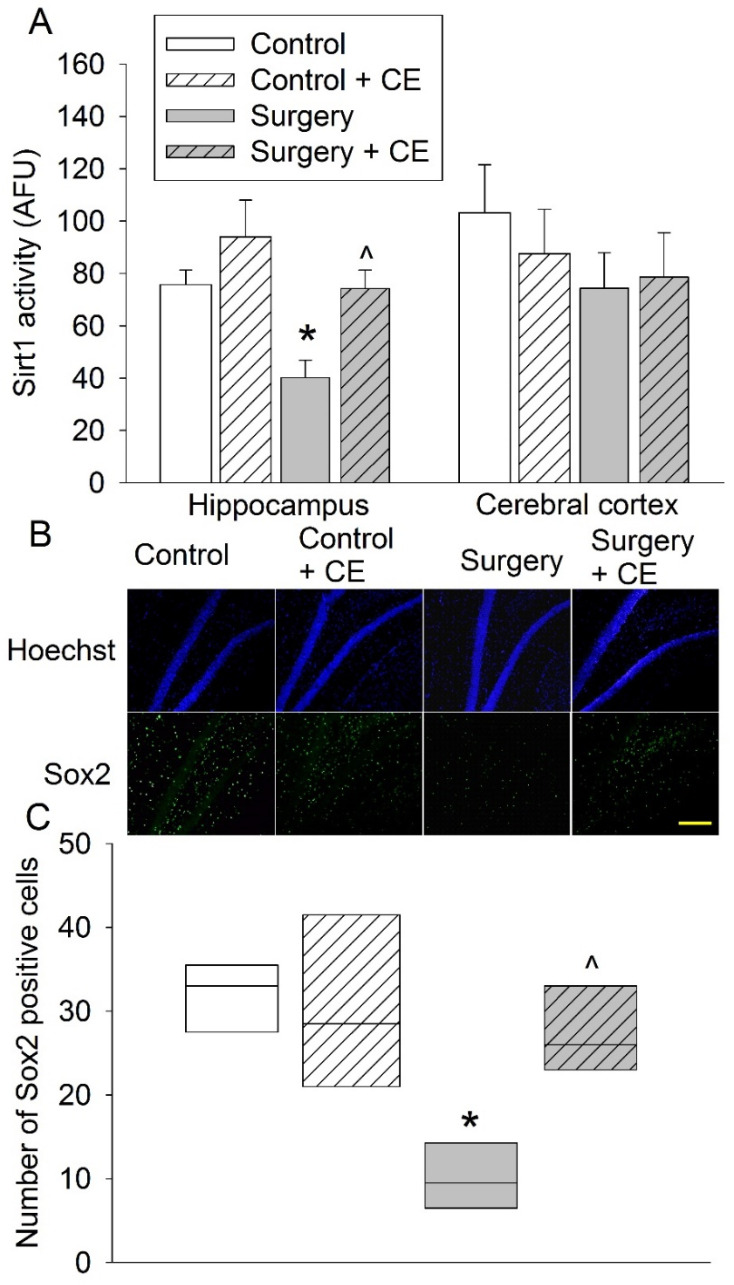
Effects of cognitive enrichment and surgery on Sirt1 activity and Sox2 expression in the brain. Eighteen-month-old mice were subjected to right carotid artery exposure and cognitive enrichment. Their brains were harvested 3 days after surgery for Sirt1 activity measurement or 2 weeks after surgery for Sox2 staining. (**A**) Sirt1 activity. (**B**) Representative images of hippocampal dentate gyrus. Scale bar: 200 µm. (**C**) Quantitative results. Results are means ± S.E.M. (*n* = 6–8). * *p* < 0.05 compared with control, ^ *p* < 0.05 compared with surgery group.

Surgery reduced Sirt1 activity in the hippocampus. This reduction was attenuated by CE. The change of Sirt1 activity in the cerebral cortex was not significant among the four experiments (Figure 7A). Similar to the results of the previous set of experiments, surgery reduced Sox2-positive cells in the hippocampus and this reduction was blocked by CE (Figure 7B,C). These results suggest that CE is effective in blocking the surgical effects on stem cells in the hippocampus.

### 3.4. Sirt1 Activation Attenuated Surgery-Induced Impairment of Learning and Memory and Reduction of Sox2-Positive Cells

**Figure 8:**
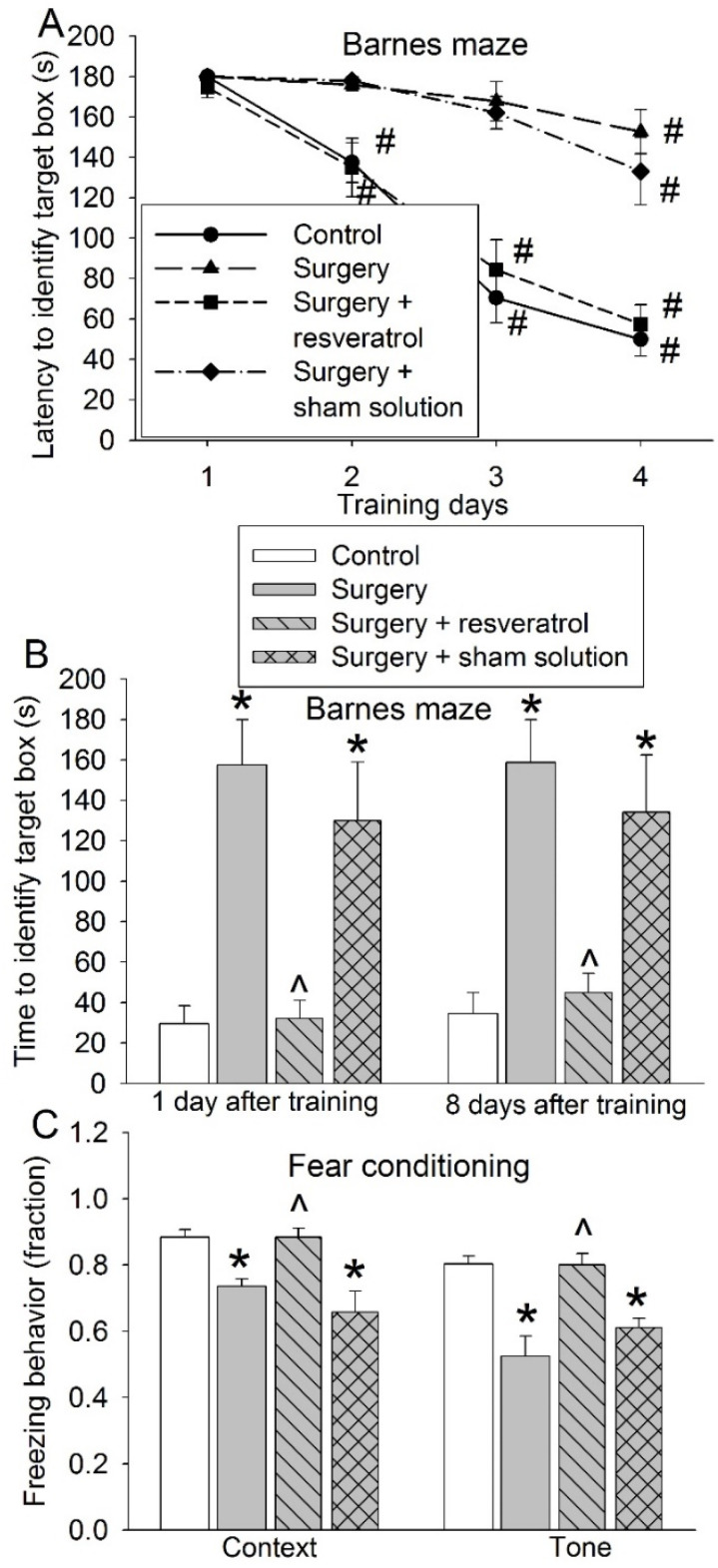
Effects of resveratrol and surgery on learning and memory. Eighteen-month-old mice were subjected to right carotid artery exposure and received resveratrol for 7 days. They started to be tested by the Barnes maze and fear conditioning 2 weeks after surgery. (**A**) Performance during the training sessions of the Barnes maze test. (**B**) Performance during the memory phase of the Barnes maze test. (**C**) Performance in the fear-conditioning test. Results are means ± S.E.M. (*n* = 9–11). # *p* < 0.05 compared with the corresponding data on day 1, * *p* < 0.05 compared with control, ^ *p* < 0.05 compared with surgery group.

**Figure 9:**
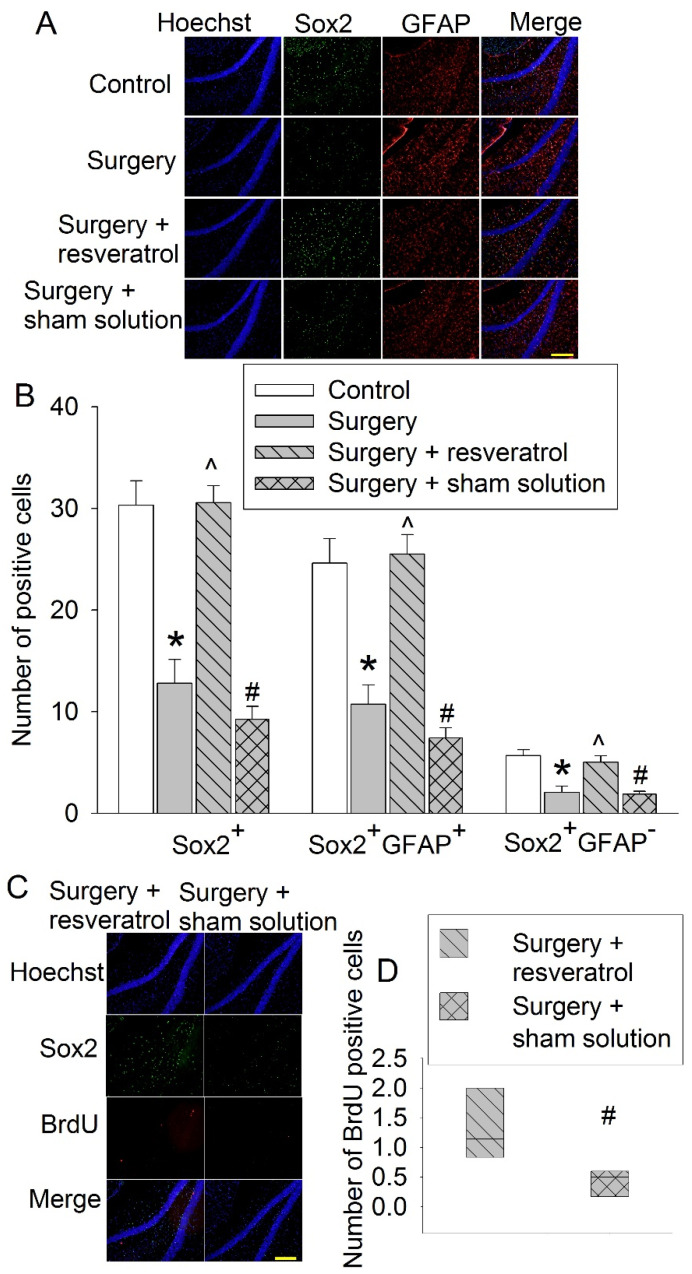
Effects of resveratrol on Sox2 expression and cell genesis in the hippocampus. Eighteen-month-old mice were subjected to right carotid artery exposure and received resveratrol for 7 days. Their brains were harvested 2 weeks after surgery. (**A**) Representative images of hippocampal dentate gyrus. Scale bar: 200 µm. (**B**) Quantitative results of Sox2-positive results. (**C**) Representative images of hippocampal dentate gyrus. Scale bar: 200 µm. (**D**) Quantitative results of BrdU-positive cells. Results are means ± S.E.M. (*n* = 6 for panel B and *n* = 7 for panel E). * *p* < 0.05 compared with control, ^ *p* < 0.05 compared with surgery group, # *p* < 0.05 compared with surgery plus resveratrol.

To determine whether the deceased Sirt1 plays a role in the surgery-induced impairment of learning and memory, resveratrol, a Sirt1 activator [20], was used. Resveratrol blocked the surgical effects on the performance of mice in the training sessions (F(1,18) = 30.771, *p* < 0.001) and memory phase of the Barnes maze tests and the context-related and tone-related fear conditioning (Figure 8). These results suggest that resveratrol attenuates surgery-induced learning and memory impairment. Resveratrol also blocked the surgical effects on the number of Sox2-positive cells in the hippocampus (Figure 9A,B). Finally, resveratrol increased BrdU-positive cells in mice with surgery (Figure 9C,D), suggesting that resveratrol increases newly formatted cells in the hippocampus. As shown in Figure 9C, some of the BrdU-positive cells were positive for Sox2 staining and some were not positive for Sox2 staining. However, due to very few cells being BrdU-positive in these old mice, it was not accurate to determine whether resveratrol increased BrdU-positive cells that were Sox2-positive or not.

## 4. Discussion

POCD affects a large number of patients and is associated with poor outcomes after surgery [2,5]. However, there is a lack of clinically practical interventions to reduce POCD. Our study showed that EE and CE applied after surgery attenuated POCD and that decreased Sox2 expression might contribute to POCD in old mice. These results suggest novel interventions and mechanisms for POCD.

Aging is an independent risk factor for POCD [1,2]. To simulate clinical situation, we used aged mice in the study. Similar to our finding in young adult mice [11], EE applied after surgery attenuated POCD in aged mice. Importantly, CE in which physical activity may not be an important component also attenuated POCD. CE may be clinically more applicable than EE for elderly patients after surgery. A previous study has shown that EE applied before surgery attenuates POCD in old rats [12]. Thus, it appears that EE can be applied before or after surgery to reduce POCD.

Sox2 has been shown to be critical for maintaining pluripotency of stem cells [15,36]. Our results showed that Sox2-positive cells were positive for GFAP, doublecortin or NeuN, biomarkers for astrocytes, and immature neurons or mature neurons [11,33,34,35], suggesting that Sox2-positive cells are involved in astrogenesis and neurogenesis. Consistent with this idea, co-localization of Sox2 with NeuN has been reported in multiple studies and Sox2 is considered to play a role in the differentiation of these neurons [37,38,39]. One novel finding of our study is that Sox2-positive cells may be critical for learning and memory. This finding is supported by a few lines of direct and indirect evidence. First, surgery reduced Sox2-positive cells in the hippocampus and impaired learning and memory of mice. EE or CE attenuated these effects of surgery. In addition, reducing Sox2-positive cells by Sox2 silencing in the hippocampus resulted in impaired learning and memory. Finally, surgery reduced Sirt1 activity and activating Sirt1 by resveratrol reduced a surgery-induced decrease of Sox2-positive cells and the impairment of learning and memory. Although it is not clear why a Sox2-positive cell decrease will impair learning and memory, we have shown that surgery impairs the genesis of brain cells and EE attenuates this impairment [11]. In addition, our current study showed that resveratrol reduced surgery-induced impairment of learning and memory and increased brain cell genesis after surgery. Brain cell genesis is known to be important for learning and memory [11,13]. The decrease of Sox2-positive cells may mean the decrease of a stem cell pool for cell proliferation, which may be a mechanism for the involvement of Sox2-positive cells in learning and memory.

Interestingly, a large number of Sox2-positive cells are GFAP-positive. GFAP is an astrocyte marker and has been shown to co-localize with Sox2 in astrocytes in the mouse brain [40]. Our results suggest that the majority of Sox2 may be astrocytes, even in the dentate gyrus of the hippocampus. It has been shown that Sox2 is needed for the maturation of astrocytes [41], which is needed for learning and memory [42]. Those Sox2-positive cells that are GFAP-negative may be the pool of stem cells, which was decreased by surgery. This surgery effect was attenuated by EE and resveratrol. These results suggest that EE and resveratrol preserve stem cells with pluripotency after surgery.

Our results showed that surgery decreased Sirt1. Sirt1 is a deacetylase [20]. Its decrease induces acetylation, nuclear export, ubiquitination, and degradation of Sox2 and, therefore, causes Sox2 to decrease [20]. Consistent with the findings that a surgery-induced Sirt1 decrease contributes to the decreased Sox2, resveratrol, a Sirt1 activator [20], attenuated a Sox2 decrease after surgery. It has been shown that mice lacking Sirt1 catalytic activity in the brain develop learning and memory impairment, decreased dendritic spine density and disrupted plasticity, suggesting that Sirt1 plays a critical role in learning and memory [43]. The previous study suggests that these effects on neural plasticity, learning, and memory may be mediated by miR-134 [43]. Our results suggest a novel mechanism for these effects—Sirt1 may regulate Sox2 expression to affect learning and memory.

Little is known about the effects of surgery and a relatively short episode of anesthesia on stem cells. However, studies have documented that isoflurane exposure for 4 h reduces neural stem cell proliferation and neurogenesis for 5 days in the neonatal rats [44]. The effects of isoflurane, a volatile anesthetic, and ketamine, an intravenous anesthetic, on human stem cell culture are variable, dependent on the time and dosages [45,46]. Our previous study has shown that the reduction of neurogenesis remained obvious even 12 days after surgery and anesthesia in young adult mice [11]. Our current study showed that anesthesia and surgery reduced the pool of Sox2-positive stem cells at 2 and 4 weeks after surgery in aged mice, a line of evidence for prolonged effects of surgery on stem cells. However, very few cells were BrdU-positive in the dentate gyrus of the hippocampus of old mice even after the injection of BrdU for 7 days. Direct assessment of neurogenesis was not performed because of the concerns of inaccuracy of the data.

We exposed animals to a combination of surgery and anesthesia, a common clinical situation. The combination of surgery and anesthesia induces learning and memory impairment in humans and animals [2,22]. Of note, anesthesia alone can also induce learning and memory impairment in animals [47,48]. However, anesthesia is mostly used for patients who have surgery and is less frequently used for radiology examinations or endoscopic procedures. The role of anesthesia in the development of POCD in humans is unclear yet. Nevertheless, volatile anesthetics can activate calcium channels in the plasma membrane to induce calcium influx and ryanodine receptors in the endoplasmic reticulum to induce calcium release. The overall effects are to increase intracellular calcium, which can induce cell injury [49]. One of the increased intracellular calcium effects is to enhance the breakdown of Sirt1 mRNA and therefore decrease Sirt1 protein expression [50]. Resveratrol blocks calcium influx via calcium channels and calcium release from endoplasmic reticulum to restore normal intracellular calcium signaling and maintain Sirt1 protein expression [50,51,52]. Thus, resveratrol should be able to block anesthetic-induced cell injury. In supporting this possibility, resveratrol attenuated sevoflurane- and nitrous oxide-induced learning and memory dysfunction in old rats [53].

Our study may have clinical implications. POCD is a significant clinical problem affecting 20 to 40% of elderly patients with various types of surgeries at the time of hospital discharge [2,5,54,55]. Non-pharmacological methods, such as EE and especially CE, have significant advantages because these methods do not have the risks of side effects of drugs and drug–drug interaction. The perioperative period has ongoing complex pathophysiological processes and involves the use of many medications. Reducing the burden of medications may not only decrease the risks of side effects of drugs, drug–drug interaction, and errors in administering drugs but also lowers the cost. Elderly patients may have problems performing physical exercise after surgery. These patients should be able to perform cognitive exercises, which are shown to be beneficial in this preclinical study. Also, we showed that resveratrol attenuated surgery-induced decrease of Sox2-positive cells and impairment of learning and memory. Resveratrol has many beneficial effects and limited side effects [56]. Resveratrol may be a suitable medication to reduce POCD if this effect is confirmed in humans.

Our research has limits. Our results suggest the role of Sirt1 in the surgical effects on Sox2-positive cells in the hippocampus. We have not determined why surgery reduces Sirt1. Also, we have not determined which types of Sox2-positive cells, the GFAP-positive or -negative cells, are important for learning and memory functions. Further studies are needed to gain these mechanistic insights. Finally, we used male old mice in the study. Female mice should be used to determine if EE and CE are effective to reduce POCD in these mice. However, it was difficult to obtain enough female old mice. Also, sex-related hormonal effects on learning and memory may be less in old mice than that in young mice because the levels of sex-related hormones are low in old mice.

Multiple directions of future studies can be planned. First, we showed that EE and CE attenuated POCD. Determining whether EE and CE reduce POCD in higher animals than rodents can be performed before clinical trials are performed. Similar studies can be done to examine the effectiveness of resveratrol. These translational studies may ultimately establish interventions for POCD. Mechanistically, studies can be performed to determine how surgery reduces Sirt1 activity.

## 5. Conclusions

We have shown that EE and CE applied after surgery reduces POCD. This effect may be mediated by maintaining stem cells with pluripotency after surgery. Reserving Sox2-positive cells may be an interventional strategy to reduce POCD and to maintain learning and memory function.

## Data Availability

Available from the corresponding author upon request.

## Abbreviations

CE, cognitive enrichment; EE, environmental enrichment; GFAP, glial fibrillary acidic protein; NT, non-targeting; POCD, postoperative cognitive dysfunction; Sox2, sex-determining region Y-box-2; Sirt1, sirtuin; SE, standard environment.
